# Polyacrylamide-Based Solutions: A Comprehensive Review on Nanomaterial Integration, Supramolecular Design, and Sustainable Approaches for Integrated Reservoir Management

**DOI:** 10.3390/polym17162202

**Published:** 2025-08-12

**Authors:** Moamen Hassan Mohamed, Mysara Eissa Mohyaldinn

**Affiliations:** 1 Petroleum Engineering Department, Universiti Teknologi PETRONAS, Seri Iskandar 32610, Malaysia; moamen_22011127@utp.edu.my; 2 Center of Flow Assurance, Institute of Subsurface Resources, Universiti Teknologi PETRONAS, Seri Iskandar 32610, Malaysia

**Keywords:** polyacrylamide, enhanced oil recovery (EOR), conformance control, sand management, nanomaterials, supramolecular polymers, harsh environments, sustainable solutions

## Abstract

Maximizing hydrocarbon recovery from mature and complex reservoirs is constrained by heterogeneity, sand production, and harsh operational conditions. While polyacrylamide (PAM)-based systems are pivotal in addressing these challenges, a comprehensive synthesis of their transformative evolution and multifunctional capabilities remains overdue. This review critically analyzes advancements in PAM-based materials for enhanced oil recovery (EOR), conformance control, and sand management. We show that nanomaterial integration (e.g., magnetic NPs, nanoclays) significantly augments PAM’s rheological control, thermal and salinity stability, interfacial properties, and wettability alteration. Furthermore, the emergence of supramolecular chemistry has endowed PAM systems with unprecedented resilience, enabling self-healing and adaptive performance under extreme subsurface conditions. The review highlights a crucial paradigm shift towards integrated reservoir management, synergizing these advanced chemical designs with mechanical strategies and leveraging sophisticated monitoring and predictive analytics. Critically, innovations in sustainable and bio-inspired PAM materials offer environmentally responsible solutions with enhanced biodegradability. This synthesis provides a holistic understanding of the state of the art. Despite persistent challenges in scalability and predictability, continually re-engineered PAM systems are positioned as an indispensable and increasingly sustainable cornerstone for future hydrocarbon recovery in the complex energy landscape.

## 1. Introduction

The escalating global energy demand, coupled with the increasing maturity of conventional hydrocarbon reservoirs, has heightened the emphasis on optimizing recovery from current fields. A significant amount of global hydrocarbon reserves, typically between 70% and 80% of the original oil in place (OOIP), is not recoverable using traditional primary and secondary techniques [1,2]. This situation highlights the essential requirement for sophisticated enhanced oil recovery (EOR) technologies to access these overlooked reserves and prolong the economic viability of aging oilfields [3,4].

The complexities of reservoir challenges often impede efficient hydrocarbon extraction. Primary among these is inherent reservoir heterogeneity, which gives rise to preferential flow paths, uneven fluid distribution, and premature water or gas breakthrough, ultimately resulting in high water-cut or gas–oil ratio issues [1]. Addressing this, excessive water production poses significant economic challenges, resulting in increased operational costs related to water treatment and disposal, which ultimately reduces project profitability [2,5]. Furthermore, the integrity of the wellbore in unconsolidated sandstone formations encounters persistent challenges due to sand production, as migrating sand particles can erode production equipment, obstruct flow paths, and result in wellbore instability. The identified issues require significant interventions and corrective actions [3,6,7,8,9]. Moreover, the drive to utilize unconventional and ultra-deep reservoirs presents challenging operational conditions, marked by extreme temperatures that may surpass 200 °C, elevated salinities reaching up to 30% total dissolved solids, and the occurrence of corrosive substances, such as H_2_S and CO_2_, which considerably impair the effectiveness of traditional EOR chemicals [2,10].

To address these widespread challenges, “conformance control” and “sand management” have become essential strategies in enhanced oil recovery. The objective of conformance control is to improve fluid flow patterns in the reservoir, thereby increasing volumetric sweep efficiency by redirecting injected fluids away from high-permeability thief zones and into unswept, oil-rich regions [1]. At the same time, sand management focuses on stabilizing weak formations to avoid the detachment and movement of sand particles, which helps maintain wellbore integrity, safeguard equipment, and ensure long-term productivity [6,7,9]. The two fundamental aspects of reservoir management play a vital role in enhancing overall oil recovery, minimizing operational expenses, and prolonging the productive lifespan of oil and gas assets.

**Figure 1:**
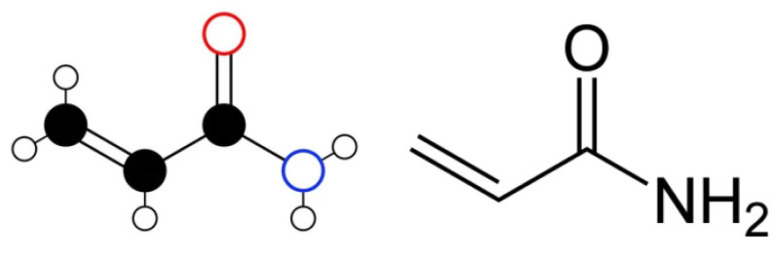
Structure of acrylamide monomer.

Central to these strategies are polyacrylamides (PAM), which are water-soluble synthetic linear polymers derived from acrylamide or a mixture of acrylamide and acrylic acid monomers [11,12]. Acrylamide (CH_2_=CH-CO-NH_2_; 2-propenamide; CAS RN 79-06-1) is a colorless, odorless white crystalline solid. It has a molecular weight of 71.08 g/mol, a melting point of 84.5 °C, and a low vapor pressure of 0.007 mmHg at 25 °C, with a boiling point of 136 °C at 3.3 kPa/25 mmHg [13]. It is highly soluble in water, acetone, and ethanol, but insoluble in nonpolar solvents. Produced industrially since the 1950s through the hydration of acrylonitrile, acrylamide is also known by synonyms such as acrylic amide or vinyl amide. The molecular structure of polyacrylamide monomer (acrylamide) is shown in Figure 1.

Consequently, PAM and its derivatives have been recognized in the petroleum industry for their versatility and cost-effectiveness [14]. Their practicality arises from their relatively low cost and their ability to be formulated to high molecular weights, frequently attaining several million g/mol [15]. Their applications extend beyond standard polymer flooding, improving mobility control through their viscosifying characteristics [4,16], to more specific roles in well interventions. The structural flexibility of PAM allows for various chemical modifications, such as hydrolysis or copolymerization with monomers like acrylamido-tertiary-butyl sulfonic acid (ATBS) or acrylic acid (AA). This capability allows for the customization of properties like thermal stability, salinity tolerance, and rheological behavior to meet various reservoir conditions [17]. This review seeks to consolidate recent progress in PAM-based systems for integrated reservoir conformance, sand management, and EOR, drawing from established knowledge to offer a modern viewpoint on the innovations propelling the field ahead.

**Figure 2:**
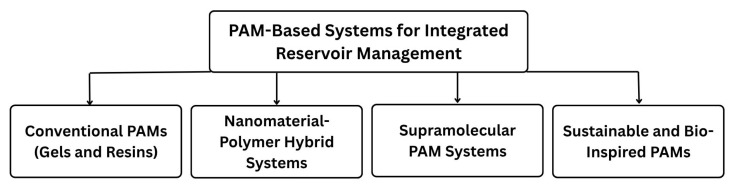
Evolution and application spectrum of polyacrylamide-based systems for integrated reservoir management.

This thorough work reviews the progression of PAM-based materials, from traditional applications to innovative technologies, as illustrated in Figure 2. It delves into the historical progression and basic classifications of PAM-based gels and early sand stabilization methods, outlining their fundamental mechanisms and inherent limitations. A significant focus is placed on advanced PAM-based materials, which include systems that integrate innovative crosslinkers, nanomaterial–polymer hybrids, and supramolecular polymers. This exploration clarifies how these advancements provide improved properties and distinct mechanisms for enhanced oil recovery and sand control, especially in demanding environments. Furthermore, the review explores the development and application of sustainable and bio-inspired PAM systems, addressing significant environmental concerns through biodegradation research and the incorporation of biomass-derived elements [5,10,18,19]. The collaborative interaction between chemical and mechanical approaches for comprehensive reservoir management, along with the significance of cutting-edge monitoring and forecasting technologies [8,20], is also extensively examined. This review synthesizes insights from a diverse array of recent literature (e.g., [21,22,23,24,25]) to provide a critical comparative analysis of material properties, mechanistic understanding, and characterization techniques. It serves as a valuable resource for those dedicated to advancing efficient, cost-effective, and environmentally sustainable solutions for complex petroleum reservoirs.

## 2. Chemistry and Applications of Key Polyacrylamide Derivatives

While the basic structure of polyacrylamide (PAM) is versatile, its performance in diverse and challenging reservoir environments is significantly enhanced through targeted chemical modifications. The primary strategy involves the copolymerization of the acrylamide monomer with various functional co-monomers, resulting in a family of derivatives. These modifications introduce new functional groups onto the polymer backbone, which profoundly alters the polymer’s charge density, thermal stability, salinity tolerance, and associative behavior in solution.

**Figure 3:**
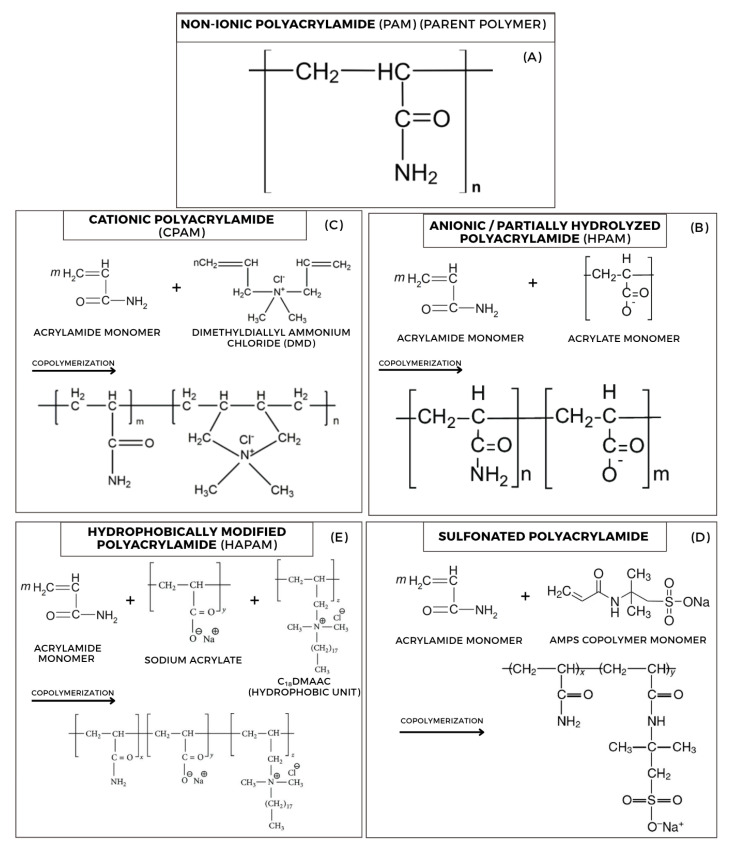
Chemical structures and synthesis of key polyacrylamide derivatives. (**A**) The structure of the non-ionic parent polymer, polyacrylamide (PAM). The primary derivatives are formed via copolymerization: (**B**) anionic/partially hydrolyzed polyacrylamide (HPAM) is a copolymer of acrylamide and sodium acrylate; (**C**) cationic polyacrylamide (CPAM) is formed with a cationic co-monomer like diallyldimethylammonium chloride (DMD); (**D**) sulfonated polyacrylamide is formed with a thermally stable co-monomer like AMPS; and (**E**) hydrophobically modified polyacrylamide (HAPAM) is a terpolymer incorporating acrylamide, anionic, and hydrophobic units.

The primary classes of PAM derivatives used in the oil and gas industry are defined by the nature of their co-monomers, with their synthesis routes and final structures illustrated schematically in Figure 3.

Anionic/partially hydrolyzed polyacrylamide (HPAM) remains the most widely deployed polymer for EOR, primarily due to its cost-effectiveness and exceptional viscosifying ability in low-salinity water [26,27]. While HPAM can be produced by post-polymerization hydrolysis of non-ionic PAM, it is commonly synthesized via the copolymerization of acrylamide and sodium acrylate, as illustrated in Figure 3B. HPAM’s key feature is the presence of anionic carboxylate (-COO^−^) groups. In low-salinity environments, these negatively charged groups induce strong intramolecular electrostatic repulsion, causing the polymer chain to uncoil and adopt a highly extended conformation. This expansion dramatically increases the polymer’s hydrodynamic volume, resulting in a significant increase in solution viscosity and a more favorable mobility ratio for displacing oil [28]. However, this reliance on charge repulsion is also its primary weakness. In high-salinity or hard brines, the abundant cations (Na^+^, Ca^2+^, Mg^2+^) effectively screen the negative charges, causing the polymer chain to collapse and lose its viscosifying power. Divalent cations are particularly detrimental, as they can form ionic bridges that lead to polymer precipitation [1,29].

Cationic polyacrylamide (CPAM) is synthesized by copolymerizing acrylamide with a cationic monomer, such as diallyldimethylammonium chloride (DMDMAC), which cyclizes during polymerization to form a stable ring structure within the backbone (Figure 3E. The resulting quaternary ammonium (-N^+^R_3_) groups impart a net positive charge to the polymer. This functionality makes CPAM unsuitable for traditional EOR mobility control (where it would strongly and undesirably adsorb to negatively charged sandstones), but makes it exceptionally effective for clay stabilization. The positive charges on the CPAM chain are strongly attracted to the negatively charged surfaces of formation clays (e.g., silica, aluminosilicates). This strong, irreversible adsorption neutralizes the clay’s surface charge and binds particles together, preventing the swelling and fines migration that cause severe formation damage, particularly during the injection of low-salinity fluids. Its other major application in the industry is as a flocculant in the treatment of oily produced water.

Sulfonated polyacrylamide represents a critical advancement for applications in high-temperature, high-salinity (HTHS) reservoirs. This derivative is a copolymer of acrylamide and a sulfonated monomer, most commonly AMPS (2-acrylamido-2-methylpropane sulfonic acid), as depicted in Figure 3D. The bulky, strongly acidic sulfonate group (-SO_3_^−^) provides two crucial advantages over the carboxylate group in HPAM. First, the C-S bond in the AMPS monomer is inherently more thermally stable than the C-N amide bond, leading to superior thermal stability at temperatures often exceeding 120 °C [30]. Second, the bulky C(CH_3_)_2_-CH_2_ group provides steric hindrance, physically shielding the anionic sulfonate group. This shielding dramatically reduces the group’s interaction with divalent cations (Ca^2+^, Mg^2+^), preventing the ionic bridging and precipitation that plague HPAM in hard brines [17,31]. Consequently, sulfonated copolymers maintain their viscosity and performance in environments where HPAM would fail.

Hydrophobically modified polyacrylamide (HAPAM) is another class of advanced polymers designed for harsh conditions, which rely on a different viscosifying mechanism. HAPAM is created by copolymerizing acrylamide with a small amount of a hydrophobic monomer that contains a long alkyl “tail” (e.g., the C_18_DMAAC unit in Figure 3E). While the polymer backbone remains water-soluble, these hydrophobic tails, in an aqueous environment, are driven by thermodynamics to aggregate into micelle-like microdomains. These clusters act as dynamic, physical cross-links between different polymer chains, creating a transient network that significantly increases solution viscosity. Because this association is driven by thermodynamics rather than electrostatics, it is far less sensitive to the presence of salts. As a result, HAPAMs exhibit robust viscosity and stability in high-salinity brines and can even display a “salt-thickening” effect [32,33,34]. Advanced designs often create multifunctional terpolymers that incorporate both hydrophobic and anionic groups into a single backbone to achieve a combination of associative and electrostatic thickening.

The key characteristics, functionalities, and applications of these fundamental derivatives are concisely summarized for comparison in Table 1.

## 3. Historical Evolution of Polyacrylamide-Based Systems

**Figure 4:**
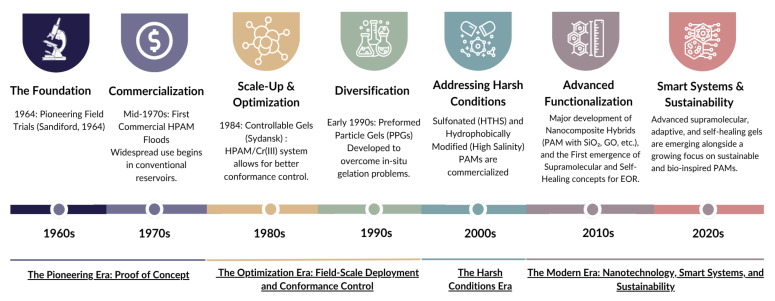
Historical timeline of key milestones in the development of polyacrylamide-based systems for oil and gas applications. The progression is marked by distinct eras: the Pioneering Era established the proof of concept for HPAM in EOR. The Optimization Era focused on large-scale deployment and the development of gels (in situ and PPGs) for conformance control. The Harsh Conditions Era saw the commercialization of advanced copolymers (sulfonated and hydrophobically modified) to overcome thermal and salinity limitations. The Modern Era is characterized by advanced functionalization through nanotechnology and the emergence of smart systems, including supramolecular, self-healing, and sustainable bio-inspired polymers.

The development of PAM reflects over a half-century of advancements in polymer chemistry and reservoir engineering. This progression, marked by distinct eras of technological focus, is illustrated in the historical timeline in Figure 4.

The Pioneering Era (1960s–1970s) established the fundamental proof of concept for polymer flooding. Following the industrial synthesis of PAM, its potential as a water viscosifier was quickly recognized. The foundational work by Pye and Sandiford [39,40] provided the first comprehensive laboratory and field demonstrations that showed that injecting a diluted solution of partially hydrolyzed polyacrylamide (HPAM) could significantly improve oil recovery. Their work established the core principle of improving the mobility ratio by increasing water viscosity, leading to a more stable displacement front and enhanced volumetric sweep efficiency. By the 1970s, this technology was being commercialized, with initial field applications targeting conventional, low-temperature (<75 °C), low-salinity reservoirs where HPAM was most effective and stable [41]. This was followed by the Optimization Era (1980s–1990s), which focused on field-scale deployment and tackling the pervasive issue of reservoir heterogeneity. The 1980s saw the widespread adoption of HPAM flooding in massive field projects, most notably in China’s Daqing oilfield, which validated the technology’s economic success on an industrial scale and provided invaluable operational data [42]. However, the success of these floods highlighted a critical challenge: premature water breakthrough due to high-permeability “thief zones.” This spurred the development of conformance control technologies. A major milestone was the invention of the highly controllable in situ gel system based on HPAM and chromium (III) acetate by Sydansk [43], which allowed for the more reliable placement of plugging gels deep within the reservoir. To overcome the inherent risks and unpredictability of in situ gelation (e.g., premature gelation, shear degradation), the 1990s saw its diversification into preformed particle gels (PPGs). These millimeter-sized, superabsorbent particles were synthesized at the surface and injected to plug large fractures and conduits, offering a more robust and controllable alternative [1].

As the industry began targeting more challenging assets, the Harsh Conditions Era (2000s) was born out of necessity. The severe performance degradation of conventional HPAM in hot (>90 °C) and saline reservoirs drove a major research push to engineer more robust polymer chemistries. This led to the successful development and commercialization of advanced copolymers. The most significant of these were sulfonated polyacrylamides, typically copolymers of acrylamide and AMPS. The bulky, stable sulfonate group provided exceptional thermal stability and tolerance to divalent cations, pushing the application window to well over 120 °C [44,45]. Concurrently, hydrophobically modified polyacrylamides (HAPAM) were developed, which used the principle of physical association between hydrophobic side chains to generate viscosity, a mechanism far more resilient to high salinity than the electrostatic repulsion of HPAM [38].

Finally, the Modern Era (2010s–present) is defined by a convergence of advanced materials science and a growing focus on sustainability. The 2010s were marked by advanced functionalization, which saw an explosion of research into nanotechnology integration. Reinforcing PAM with nanoparticles like silica, alumina, or graphene oxide created robust nanocomposite hybrids with significantly enhanced stability and novel functionalities [4,46]. This decade also saw the first serious application of supramolecular and self-healing concepts from materials science to EOR challenges. Building on this foundation, the 2020s have focused on smart systems and sustainability. This includes the maturation of advanced supramolecular gels that can adapt to reservoir stimuli and self-heal after damage, and a critical focus on developing sustainable and bio-inspired PAMs (e.g., using lignin or other biomass) to address the full life-cycle and environmental impact of oilfield chemicals [2,47]. This ongoing evolution, from a simple viscosifier to a platform for intelligent materials, underscores the enduring importance and adaptability of polyacrylamide in maximizing the world’s hydrocarbon resources.

## 4. Conventional PAM Gels for Conformance Control and Early Sand Stabilization

The evolution of chemical treatments in the oil and gas industry is rooted in a need for cost-effective and adaptable solutions to enhance sweep efficiency and mitigate production challenges. Early efforts primarily focused on in situ systems designed to create barriers within the reservoir, gradually diversifying to preformed materials and dedicated sand stabilization agents.

### 4.1. In Situ Monomer-Based Gels

The earliest applications of PAM in conformance control involved in situ monomer-based gels, which are primarily composed of acrylamide monomers (structure shown in Figure 1). The fundamental principle behind these systems was to inject a low-viscosity monomer solution, typically with an initial viscosity comparable to water (1–1.3 cP), to ensure deep penetration into various reservoir zones [1]. After placement, an in situ polymerization process triggered by free radicals would create a gel. The inability to regulate the gelation time, which mostly resulted in premature gelation, was a major disadvantage. Under different reservoir temperatures, this control was difficult [1]. To counter premature gelation, retardants like potassium ferricyanide were frequently added. Research on similar acrylate-based grouting materials, which also undergo in situ polymerization, highlights that gelation time can be precisely controlled by adjusting the concentration of accelerators and initiators [48]. For instance, it has been demonstrated that increasing initiator content consistently reduces setting time, whereas accelerator content has a more complex effect, first decreasing and then increasing setting time [48]. Concerns regarding the neurotoxicity and suspected carcinogenicity of acrylamide [1,48] as well as the high monomer concentrations (4–10%) needed for adequate gel strength, made these systems less popular despite their deep penetration potential [1].

### 4.2. In Situ Polymer-Based Gels

**Figure 5:**
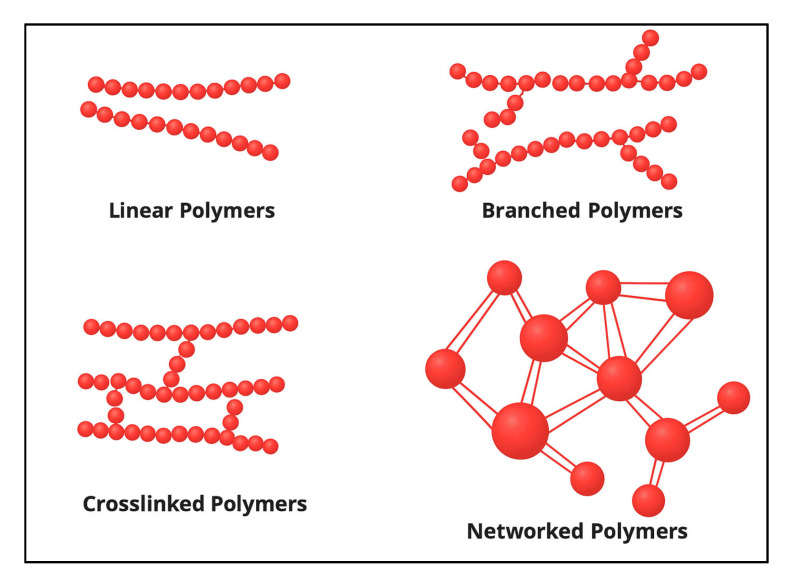
Types of polymer networks: linear, branched, crosslinked, and networked.

In situ polymer-based gels, which developed on the early monomer systems, employed diverse crosslinkers and polyacrylamide and its derivatives (such as HPAM); Figure 3 shows their structures. These systems aimed to establish a three-dimensional polymer network within the reservoir by chemically or physically linking adjacent polymer molecules, forming a continuous semisolid 3D structure that can comprise linear, branched, or heavily crosslinked polymer chains. These various configurations of polymer networks are illustrated in Figure 5.

Initially, HPAM cross-linked with aluminum citrate was pioneered in the 1970s [49] and developed and commercialized in the 1980s. While allowing surface mixing of acidic solutions for successful injection, gelation occurred rapidly and uncontrollably once pH increased within thme formation, limiting their application to near-wellbore water shutoff as permeability blockers. To achieve better control, Cr(VI) cross-linking agents were introduced. Cr(VI) is inert but converts to cross-linking Cr(III) via a redox agent upon injection, theoretically allowing deeper placement before gelling. However, these systems were sensitive to high temperatures, H_2_S interference, and posed carcinogenic risks due to Cr(VI) [1,50,51], leading to their declining use in favor of safer and more controllable alternatives.

A significant breakthrough came in 1984 with the development of the HPAM/Cr(III) acetate gel system by Sydansk. This system offered controllable gelation times, exhibited insensitivity to a wide pH range (2–12.5), and demonstrated improved resistance to common oilfield interferences. It could be applied in high-temperature reservoirs, even exceeding 124 °C [52]. However, challenges persisted, including the formation of pre-gel aggregates susceptible to reservoir filtration, which could block flow paths and hinder deep propagation, and differential adsorption rates of the polymer and cross-linker, leading to downhole ratio variations that impacted conformance control effectiveness [1,29,53]. For instance, HPAM’s hydrolysis rate, a primary degradation mechanism, significantly increases with both temperature and the presence of brine, leading to substantial viscosity loss (50% viscosity reduction at 58 °C over two years) [17,29]. Variants like colloidal dispersion gels (CDG) were also developed, utilizing low concentrations of polymer and cross-linker for cost-effectiveness. While claimed to propagate significant distances into thief zones (several Darcies permeability), laboratory tests confirmed their penetration was largely limited to extremely high-permeability channels [54]. Sahoo et al. [16] demonstrate a specific PHPA gel with Cr(III) acetate in the context of biogas purification, noting its reliance on the stability and rheological behavior of this conventional in situ PAM gel system, highlighting PAM’s versatility beyond EOR.

For harsh reservoir environments, organically cross-linked PAM systems were developed to overcome the limitations of metallic crosslinkers. Phenolic compounds like phenol and resorcinol cross-linked with formaldehyde created gels stable at temperatures up to 149 °C or higher (marketed as Flowperm325), though these required a pH greater than 9 and were sensitive to salinity [55]. Subsequent developments included sulfomethylated resorcinol-formaldehyde (SMRF) systems, which extended gelation time and allowed gelation across a wider pH range (as low as 5), despite persistent toxicity concerns for organic crosslinkers [56,57]. Hydroquinone (HQ) and hexamethylenetetramine (HMTA) combinations were designed for even higher temperatures (up to 176.7 °C) with gelation times of several days, utilizing secondary crosslinkers (e.g., terephthalaldehyde) to stabilize gels. Cost-effectiveness remained a barrier to widespread field application. Newer systems, such as acrylamide/t-butyl acrylate copolymers cross-linked with polyethyleneimine (PEI), offered polymer-controlled cross-linking delay, excellent propagation, and thermal stability up to 148.9 °C [1]. However, some studies found PEI-crosslinked gels to have lower gel strength than chromium or phenolic crosslinkers [20]. The thermal degradation of HPAM, a core component of these in situ gels, is further characterized by Beteta et al. [17], highlighting the influence of acrylic acid content and temperature on viscosity retention and hydrolysis, critical for long-term stability in these systems.

### 4.3. Preformed Particle Gels (PPG)

To circumvent the inherent drawbacks of in situ gel systems—such as uncontrollable gelation time, shear degradation, chromatographic fractionation, and dilution by formation water—preformed particle gels (PPG) emerged as a promising alternative. These gels are synthesized and prepared as particles at surface facilities before injection, offering improved control over their properties and simplified injection procedures. Micrometer-sized particle gels (microgels) operate by plugging pore throats through bridging mechanisms, where microgels form near pore constrictions and grow into a thick gelled surface layer by associating with adsorbed macromolecules. pH-sensitive microgels (e.g., Carbopol^®^) are injected under acidic conditions, where polymer molecules remain tightly coiled, resulting in low viscosity and ease of injection. Upon contact with higher-pH formation minerals, the chains uncoil and swell by absorbing formation water, leading to a significant viscosity increase and effective permeability blocking. However, their propagation depth can be limited by filtration retention in mid-permeability rocks, generally suitable for extremely high-permeability zones [1].

Millimeter-sized preformed particle gels (PPG) are typically prepared through solution polymerization followed by crushing and sieving to desired sizes. These dry particles are designed for strength, size control, environmental friendliness, and insensitivity to reservoir minerals and water salinity. After swelling in water, PPGs propagate through porous media via mechanisms including particle deformation, shrinking due to water expulsion, and particle breakage. Their application is primarily limited to reservoirs with induced fractures or large fracture-like channels due to their relatively large, instantly swelling nature [1]. Marandi et al. [22] provide experimental validation for PAM hydrogels for sand control, demonstrating significant reductions in sand production (90%) and disproportionate permeability reduction (DPR), with water permeability decreasing 95 times more than oil permeability (7.5 times). This emphasizes the utility of controlling gel properties pre-injection for specific field applications like sand control and water management.

Submicro-sized particle gels (Bright Water™) represent another class of PPGs, typically composed of cross-linked sulfonate-containing PAM microparticles with both labile and stable cross-linkers. A key feature is their thermo-responsive property: as reservoir temperature increases, labile cross-linkers de-crosslink, allowing the particles to expand aggressively by absorbing more surrounding fluids, while stable cross-linkers maintain the gel network. These systems are designed for in-depth fluid diversion in hundreds of millidarcy zones. Despite their innovative design, widespread utilization has been limited by concerns over their thermal stability and a narrow optimal application range.

### 4.4. Early Chemical Sand Stabilization (Resins)

Concurrent with the development of PAM gels, early chemical sand stabilization methods, primarily utilizing resins, emerged for chemical consolidation as an alternative to mechanical sand control techniques. The core principle involved injecting reactive chemical solutions into unconsolidated formations to bind loose sand grains together, forming a strong, permeable matrix [6,7,9]. This process prevents the dislodging of sand particles by fluid drag forces, thereby increasing the compressive strength of the formation and maintaining wellbore integrity. Figure 6 from [58] study presents a schematic diagram illustrating the process

Various resin types, including epoxy, furan, phenolic, urea–formaldehyde, acrylic, and vinyl ester resins, have been employed [6,7,9]. These resins are typically injected as liquid solutions which then polymerize and harden in situ, often facilitated by catalysts or curing agents that accelerate the reaction [9]. Key desired properties include maintaining rock permeability by ensuring the resin only bridges grains rather than completely filling pore spaces, and achieving a high compressive strength, with some hydrogels demonstrating significant increases in strength [22].

Early resin treatments offered several advantages, including the creation of durable consolidated zones and potentially lower costs compared to mechanical methods. They could also be compatible with existing gravel packing systems. However, significant limitations quickly became apparent: resins often reduced oil permeability by occupying pore space, had variable effectiveness due to permeability anisotropy leading to non-uniform distribution, and posed environmental/toxicity concerns. Premature hardening during injection and disposal challenges were also notable drawbacks [6,9]

Commercial resin-based systems like Halliburton’s SandTrap ABC (utilizing proprietary filter media) and Champion SC2020 demonstrated efficacy in high-sand production environments [9]. Additional examples of this kind of treatment are SandAid from Lubrizol and Sand Stop Aqua from Baker Hughes, which is a water/polymer mixture that creates a gel-like filter cake that has the ability of self-healing. These systems differ in their chemical composition and activation techniques, which impacts their performance in various well completions. Mörtl [6] discussed testing fluid loss additives and water-based resin systems to determine whether they could be employed as diverting agents in sand consolidation. In terms of fluid loss and ease of use, he likened them to PAM gels. This study demonstrated the significance of properties such as spurt loss and filter cake characteristics for the proper placement of resin and the removal of fluids from the wellbore.

## 5. Nanoparticle-Enhanced PAM Systems

**Figure 7:**
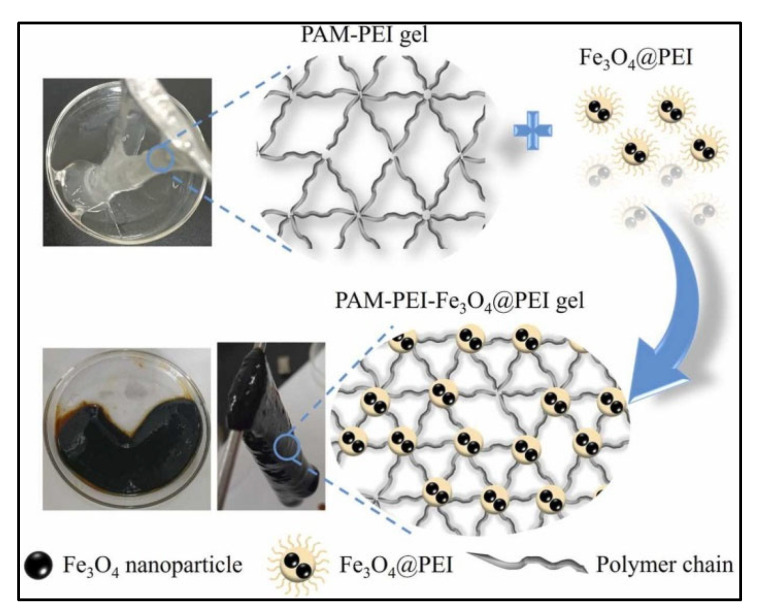
Schematic diagram of the mechanism of Fe3O4@PEI nanoparticle modulation of PAM-PEI gel structure and properties from [59].

The intrinsic limitations of conventional PAM, especially its inadequate stability under extreme reservoir conditions, led to the development of novel, multifunctional materials. Among the most promising advances are nanomaterial–polymer hybrids, which amalgamate the distinctive properties of nanoparticles to markedly enhance PAM-based systems for enhanced oil recovery [4]. This section examines the composition and mechanics of these advanced hybrids, emphasizing their practical contributions to sand management, compliance control, and the overall efficacy of EOR projects. Figure 7 depicts several of the functions of nanoparticles.

### 5.1. PAM–Nanoparticle Gels

The development of polymer gel systems, characterized by improved structural integrity, thermal stability, and responsiveness, is a primary objective, with advanced nanoparticles assuming an increasingly significant role [60]. Magnetic PAM gels represent a significant advancement in the field. These systems, integrated with iron oxide (Fe_3_O_4_) nanoparticles, exhibit adjustable properties and respond accurately to external magnetic fields.

A recent study by K. Wang et al. [59] presented the development of a core–shell Fe_3_O_4_@PEI (polyethyleneimine) nanoparticle, intended as an advanced crosslinker for PAM-PEI gels. This design successfully merges the basic inorganic stability of the iron oxide core with the chemical reactivity of the PEI shell, facilitating robust transamidation cross-linking with PAM chains. In stark contrast to conventional HPAM/Cr(III) gels, which begin to degrade and lose structural integrity around 120–140 °C, the smart polymer gels in [59] exhibited a solid-like state at 120 °C, displaying significant mechanical strength (storage modulus of 25,490 Pa). Further advancements with nanosheets [18] elevated the thermal decomposition temperature to 198.45 °C, an almost 50 °C improvement over the upper tolerance of even robust conventional organic cross-linked gels (~150 °C). This thermal resilience considerably exceeds the typical ~124 °C limit of conventional HPAM/Cr(III) gels. Figure 7 shows an illustration of this process and how the polymer chain looks against the polymer chain with the integration of nanoparticles.

In a different application of this principle, Fouji et al. [61] demonstrated that using an external magnetic field to align Fe_3_O_4_ NPs into “columnar structures” could dramatically boost oil recovery (from 29.0% to 44.6% in carbonate and 40.09% to 77.72% in sandstone cores) by acting as microscopic pistons. The broader concept of using tailored nanoparticles extends beyond magnetic materials; for instance, Kalgaonkar et al. (2019) [62] showed that surface-modified silica nanoparticles could be precisely tuned for either water shutoff (resulting in 1% regain permeability) or sand control (achieving 45% regain permeability) at temperatures up to 149 °C by controlling their surface chemistry.

In addition to magnetic gels, functionalized magnetite nanoparticles (Fe_3_O_4_) are another attractive method for creating nanocomposite hydrogels. These efficiently bridge polymer chains by acting as direct inorganic crosslinkers. Researchers have repeatedly verified a consistent distribution of these minuscule, 5–14 nm nanoparticles throughout the polymer matrix through in-depth microstructural analysis using SEM and HR-TEM. This precise arrangement fosters a genuinely hybrid network, seamlessly blending both organic and inorganic phases. The result is a material that demonstrates enhanced mechanical properties relative to conventional polymer gels [63].

Nanoclay–PAM systems also represent a significant development in improving the efficacy of polymer fluids. They represent a significant breakthrough in improving the efficacy of polymer fluids. Cheraghian et al. [64] illustrated the beneficial impact of clay nanoparticles on the rheological properties of PAM solutions, noting an increase in viscosity within a specified concentration range. Cheraghian et al. (2016) [65] carried out a follow-up study that demonstrated the considerable influence of adding nanoclay (Na-Mt) to HPAM solutions on fluid interactions. The findings were evidenced by a swift decrease in the zeta potential of oil droplets, a slight decrease in interfacial tension (IFT), and an enhancement in dilational viscoelasticity. Cheraghian et al. (2016) [65] illustrated that concentrations of Na-Mt surpassing 250 mg/L were significantly effective, promoting the formation of stable oil–mineral aggregates, a vital mechanism for improving produced water treatment from polymer flooding. The study demonstrated that nanoclays have the potential to mitigate the adverse impacts of salinity on the viscosity of PAM fluid. This aligns with findings from Giraldo et al. (2013) [66], who showed that adding silica nanoparticles to an HPAM solution in a hard brine not only prevented viscosity loss, but actually increased its viscosity by 45% compared to the polymer alone, directly counteracting the severe ‘salting-out’ effect. The ability of these nanoclay hybrids to preserve crucial rheological properties in saline environments makes them an attractive option for improving EOR performance under difficult conditions. A summary of key case studies on nanoparticle-enhanced PAM systems is provided in Table 2.

### 5.2. Nanoparticle–Polymer-Surfactant (NPS) Formulations

Moving beyond standalone gels, nanoparticles are increasingly incorporated into intricate fluid systems blending polymers and surfactants (SP). The objective is to create multifunctional agents crucial for enhanced oil recovery (EOR), and these nanoparticle–polymer–surfactant (NPS) formulations have, indeed, shown considerable promise across various applications, even extending into broader scientific domains, such as colloidal engineering.

A particularly cutting-edge hybrid system in this field harnesses multiwalled carbon nanotubes (mMWCNT) in surfactant–polymer (SP) flooding. Pandey et al. [23] notably showcased the significant enhanced oil recovery potential of functionalized mMWCNTs within SP flooding. The findings of their study indicated that these nanotubes significantly decrease surfactant adsorption on rock surfaces. This is mainly accomplished by transporting surfactant molecules to the oil–water interface through Brownian motion. This mechanism significantly reduces surfactant loss, a crucial economic concern in surfactant polymer flooding. Moreover, mMWCNTs demonstrated efficacy in modifying rock wettability and improving the rheological characteristics of the SP solution, resulting in a cumulative oil recovery of approximately 70% in comparison to traditional SP or waterflooding methods alone.

The advancement of low-salinity water (LSW)–NP–polymer fluids indicates significant potential for future applications. Saw et al. (2023) [24] presented a complex fluid based on LSW that integrates PAM and SiO_2_ nanoparticles. This system utilizes the synergistic effects of LSW, which enhances oil recovery via pH increase, fine migration, EDL expansion, and multi-ion exchange; PAM, which aids in mobility control; and NPs, which improve stability and interfacial properties. The findings demonstrated that LSW–NP–polymer fluids necessitated reduced polymer concentrations to attain target viscosity in comparison to traditional formation water systems. Furthermore, they demonstrated notable decreases in interfacial tension (IFT) and advantageous changes in wettability. The synergistic combination enhanced incremental oil recovery by 13.3% of OOIP relative to LSW alone and by 22.0% of OOIP compared to FW flooding in the secondary stage, establishing it as an effective and cost-efficient EOR method.

In the context of EOR, the integration of silica nanoparticles with surfactants and polymers results in synergistic effects such as decreased interfacial tension, improved wettability, and increased emulsion stability. This combination may achieve an increase in oil recovery of up to 28% after conventional water flooding, especially in high-permeability cores [4,64]. Quantitative analyses reveal that these nanofluids exhibit a higher zeta potential, reaching values of up to −39.6 mV, indicating improved colloidal stability. The incorporation of polymers improves this stability. Investigations into thiol surfactants concerning silver nanoparticles revealed enhanced emulsion stability and reduced interfacial tension with paraffin oil, underscoring the significant impact of nanoparticle–surfactant interactions on surface activity [68]. Gold nanoparticles with mixed polymer brushes of polystyrene and polyethylene glycol (PEG) have been demonstrated to act as effective surfactants in various contexts. They position themselves at oil–water interfaces, reducing oil droplet size during polymerization and exhibiting control over colloidal properties [69]. The self-assembly of polymer-based surfactants, including polyethylene oxide with nanoparticles at air–water interfaces, is crucial for dispersion stabilization and the development of novel 2D materials, highlighting the significance of surface energy and molecular interactions on material performance [70]. However, the development of these formulations also necessitates careful consideration of their environmental and biological impact; for example, toxicological studies on silver nanoparticle formulations using various stabilizers have shown significant impacts on zebrafish embryos, with cell viability ranging broadly from 6% to 100%, underscoring the need for careful formulation to minimize cytotoxicity [71]. This synergistic combination has also found utility in other oil and gas activities, such as enhancing cutting transportation in drilling [72,73].

### 5.3. Mechanisms of Nanoparticle Interaction and Contribution

**Figure 8:**
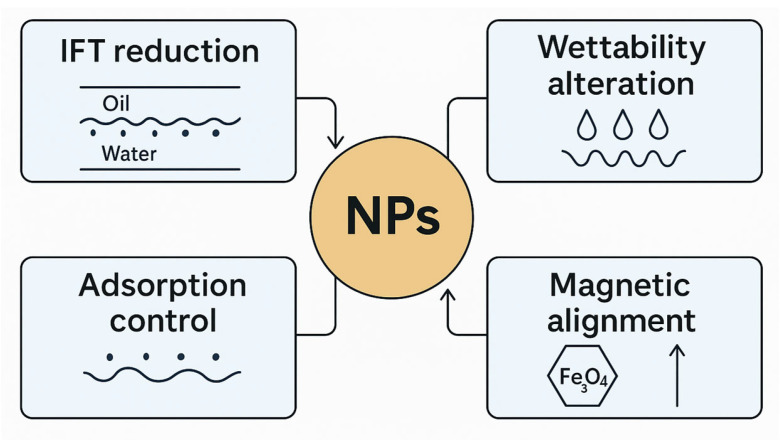
Multifunctional roles of nanoparticles in enhancing PAM-based systems.

The remarkable improvements observed in nanomaterial–polymer hybrid systems stem from various synergistic mechanisms, summarized in Figure 8, critically influencing both fluid–rock and fluid–fluid interfaces. These interactions fundamentally alter the physicochemical properties of the EOR fluid and its engagement with the reservoir system, leading to enhanced performance in multiple aspects.

Economically speaking, a major hurdle in chemical EOR is the loss of injected agents through adsorption and retention on reservoir rock surfaces, which frequently leads to significant material loss. Nanoparticles effectively combat this. They minimize polymer or surfactant adsorption either by competitively occupying surface sites or by subtly altering the rock’s surface charge, thereby making it less appealing to the injected chemicals. Consider the work by Pandey et al. (2024) [23], in which functionalized multiwalled carbon nanotubes (mMWCNT) were found to significantly reduce surfactant retention on rock surfaces, chiefly by transporting surfactant molecules directly to the oil–water interface via Brownian motion. In a similar vein, studies involving low-salinity water (LSW)–nanoparticle–polymer fluids documented reduced polymer consumption, attributable to markedly decreased adsorption compared to traditional formation water systems. When examining adsorption kinetics, HPAM on calcium carbonate (CaCO_3_) often adheres to pseudo-second-order kinetics, indicating a strong dependence on adsorbate concentration [21]. Intriguingly, higher-molecular-weight HPAM tends to exhibit greater equilibrium adsorption; for instance, F3630S (18–20 MDa) achieved the highest adsorbed amount on CaCO_3_, approximately 0.43 mg/m^2^, surpassing F3330S (11–13 MDa) at about 0.24 mg/m^2^ [74]. Salt ions introduce a nuanced dynamic. While their screening effects can diminish electrostatic attraction, thereby decreasing HPAM adhesion (e.g., F3330S adhesion dropped to 72–120 pN at 3% NaCl from its 0.1% NaCl range of 450–625 pN), salt ions can also surprisingly form “salt bridges” that, in certain cases, increase adhesion for neutral PAMs. This is particularly true with anions possessing larger bare radii [74,75]. This intricate interplay between charge screening and salt bridging ultimately governs the net adsorption behavior in saline environments. Data from core flood experiments indicate that polymer adsorption in virgin porous media, conforming to a Type IV isotherm, increases with concentration (ranging from ~22.7 µg/g at 100 ppm to ~208 µg/g at 2000 ppm). Conversely, readsorption on previously saturated cores tends to be lower, with cumulative readsorption reduced by up to 61% for a 1250 ppm polymer [76]. Optimizing oil recovery fundamentally relies on minimizing chemical loss, thereby ensuring that a larger proportion of injected agents remain active in the mobile phase.

Beyond just managing adsorption, nanoparticles notably enhance wettability alteration, a fundamental property crucial for fluid distribution and oil recovery in porous media [64,77]. Nanoparticles perform this by adsorbing onto the rock surface, effectively creating a novel nanotextured interface with modified wetting characteristics [64,78]. A key underlying mechanism here is “structural disjoining pressure.” This pressure, stemming from the organized arrangement of nanoparticles within the ultra-thin fluid film (or wedge-film) that forms between an oil droplet and the solid surface, actively pushes the aqueous phase to spread more effectively, thus promoting the detachment of trapped oil droplets. Quantitative evidence strongly supports this effect; for example, mMWCNT inclusion reduced contact angles from 57° to 36° at a 500 ppm concentration, signaling a distinct and favorable shift toward water-wet conditions [23,24]. Similarly, nanoclay additions to HPAM solutions have been observed to influence wettability by rapidly decreasing the zeta potential of oil droplets and increasing dilational viscoelasticity, which facilitates the formation of stable oil-mineral aggregates when Na-Mt concentrations exceed 250 mg/L [65]. This collective action ultimately boosts waterflooding efficiency by enabling the displacing fluid to contact and mobilize bypassed oil more effectively.

Furthermore, nanoparticles prove exceptionally adept at reducing interfacial tension (IFT) between crude oil and the displacing fluid. This serves as a key factor in mobilizing trapped oil and markedly improving microscopic displacement efficiency [24]. Nanoparticles adsorb at the oil-water interface, thereby reducing Gibbs free energy and facilitating the deformation of oil droplets. The interaction with surfactants is essential; nanoparticles act as carriers, efficiently delivering surfactant molecules to the interface, thereby improving their effectiveness [23]. Modified multiwalled carbon nanotubes (mMWCNT) have demonstrated notable reductions in IFT, achieving a decrease of up to 56% at a concentration of 100 ppm in surfactant–polymer fluids [23,61]. At the same time, fluids composed of low-salinity water (LSW) combined with nanoparticles and polymers have shown a further decrease in interfacial tension (IFT) compared to the effects of LSW by itself [24]. Reductions in IFT are crucial for reducing the capillary forces that trap oil within porous media, leading to significantly enhanced oil recovery. The combination of nanoparticles and surfactants results in a 19% enhancement in oil recovery relative to the use of surfactants alone [65].

This discussion shows that nanoparticles significantly affect rheology modification and viscoelasticity, which are essential for enhancing fluid injectivity, mobility control, and sweep efficiency in enhanced oil recovery operations. The addition of nanoparticles generally results in an increase in the viscosity of PAM solutions, which is often accomplished by promoting macromolecular structuring via hydrogen bonding or by physically entrapping nanoparticles within the polymer network. Magnetic PAM gels containing Fe_3_O_4_@PEI nanoparticles demonstrated significantly improved initial and gelation viscosities, a phenomenon linked to increased crosslinking sites and structural reinforcement [59]. In addition to viscosity, these hybrid systems often demonstrate enhanced viscoelastic properties, marked by an increased storage modulus (G′) in comparison to the loss modulus (G″). This signals a more elastic, solid-like behavior, which is crucial for mobilizing trapped oil from intricate pore spaces [22,61]. Such enhanced elasticity fosters a more efficient, piston-like displacement front, thereby mitigating viscous fingering that often bypasses oil. Nanoclay additions, for example, have been shown to improve the pseudoplasticity of polymer solutions, even with as few as 0.1 wt% of nanoparticles, and they effectively preserve rheological properties under high-salinity conditions [65].

Despite their immense potential, the field application of nanomaterial–polymer hybrid systems faces several challenges. Key among these are stability issues, including the tendency of nanoparticles to cluster or agglomerate in complex brines, leading to inhomogeneous solutions that negatively impact functionality and injectivity. Cost-effectiveness remains a concern, particularly for more advanced nanomaterials like carbon nanotubes, which have higher production costs. Ongoing research gaps include long-term stability under dynamic reservoir conditions, the potential environmental impacts of large-scale deployment, and the necessity for advanced numerical simulators that can accurately model intricate NP–fluid–rock interactions across various scales. Confronting these issues is essential for effective field-scale execution.

## 6. Adaptive Polymer Networks for Harsh Environments: Supramolecular and Dynamic Covalent Approaches

The increasing exploitation of unconventional and ultra-deep reservoirs, defined by their extreme temperatures (>200 °C), high pressures, notable salinities, and strong shear forces, renders standard polyacrylamide (PAM) systems inadequate [2]. Conventional PAMs, which feature stable covalent bonds, are susceptible to irreversible thermal degradation, mechanical scission, and salt-induced coiling, contributing to a permanent loss of functionality [1,17]. Advanced polymer designs leveraging dynamic and reversible chemistries offer a groundbreaking strategy to overcome these limitations. These approaches provide PAM-based materials with the capacity for molecular-level adaptation, enabling self-assembly, self-healing, and responsive modifications to environmental shifts.

**Figure 9:**
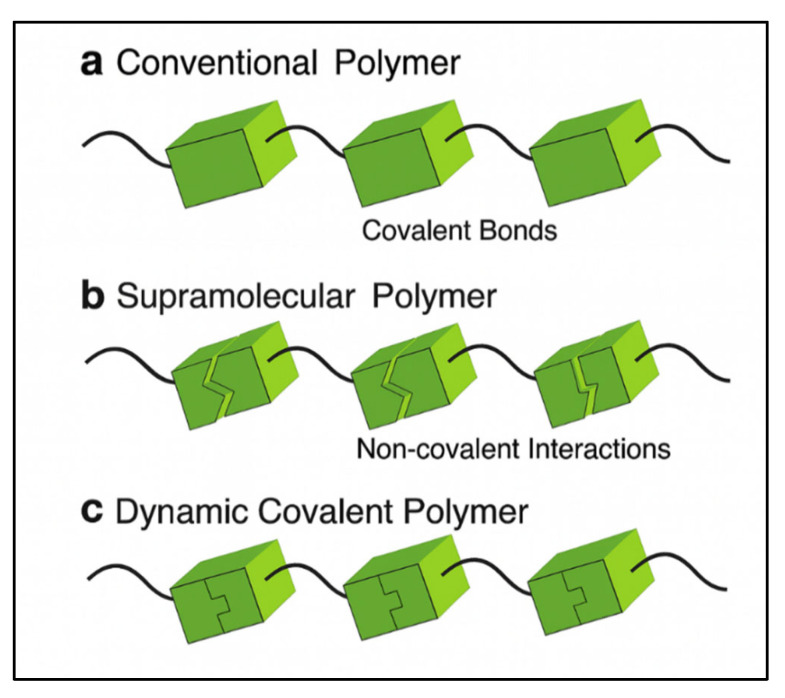
Conceptual figure of different polymer architectures. (**a**) Conventional polymers are formed by strong, irreversible covalent bonds. (**b**) Supramolecular polymers are assembled through dynamic and reversible non-covalent interactions. (**c**) Dynamic covalent polymers are linked by reversible covalent bonds, combining covalent robustness with a dynamic character) [81].

Within this domain of adaptive materials, it is crucial to distinguish between two primary mechanistic classes, the conceptual differences in which are illustrated in Figure 8. Conventional polymers (Figure 9a) are characterized by monomeric units linked via strong, static covalent bonds, resulting in a fixed and irreversible molecular structure. In contrast, supramolecular polymers (Figure 9b) are formed through the dynamic association of monomers via weaker, non-covalent interactions such as hydrogen bonding, hydrophobic association, and host–guest recognition. The discontinuous representation in the schematic highlights the transient and reversible nature of these physical crosslinks, which allows for adaptive properties but can be susceptible to environmental changes. Bridging these two classes are dynamic covalent polymers (Figure 9c), which utilize reversible covalent bonds. This is visualized by interlocking units that possess the strength of a covalent linkage but can be controllably disconnected and reconnected. This design paradigm imparts both the structural robustness of conventional polymers and the adaptive, reorganizable character of supramolecular systems. While both adaptive classes provide unique properties, their differing bond energies and activation mechanisms fundamentally differentiate their mechanisms of action, greatly exceeding the resilience of their covalently linked predecessors in demanding conditions [79,80,81].

### 6.1. Supramolecular PAMs Based on Physical Crosslinking

Supramolecular polymers are meticulously designed assemblies leveraging specific non-covalent forces to create ordered, functional, and physically cross-linked structures. The dynamic and reversible nature of these physical crosslinks allows the material to reconfigure and self-repair, overcoming the critical limitations of conventional polymers in extreme reservoir conditions. Recent research provides compelling quantitative evidence of the performance enhancements achieved through supramolecular design. One prominent strategy involves hydrophobic association, where a supramolecular gel was prepared via the micellar copolymerization of acrylamide with octadecyl methacrylate. This physically crosslinked network exhibited exceptional mechanical robustness, withstanding a compressive stress of 3.82 MPa at 88.3% compression without failure. This performance far exceeds that of typical brittle conventional PAM gels and enabled a plugging rate greater than 95% in core flood tests, demonstrating its practical utility [82].

Besides hydrophobic associations, another powerful approach utilizes electrostatic interactions. A recent work conducted by Huang et al. (2024) [83] developed a supramolecular system by mixing cationic and anionic polyacrylamides. The resulting polyelectrolyte complex demonstrated a profound increase in material efficiency; a 0.4 wt% solution of the supramolecular system achieved a viscosity and viscoelasticity comparable to a 0.6 wt% solution of a conventional polymer. The dynamic nature of the system was an evident in its superior shear resilience, retaining 73.3% of its viscosity after high-shear cycling, compared to just 53.5% for the traditional polymer. This highlights the capacity for autonomous network reformation, a key advantage over covalently crosslinked or entangled systems. Hydrogen bonding also serves as a critical tool for creating adaptive networks. Niu et al. (2024) [84] developed a gel for heavy oil reservoirs where poly(vinyl alcohol) (PVA) was incorporated as a non-covalent, hydrogen-bonding crosslinker within a polyacrylamide network. This supramolecular design imparted significant stability, achieving a temperature tolerance of 120 °C and a high-pressure blocking strength of 5.96 MPa in a fractured core. This demonstrates how targeted, non-covalent interactions can build materials that withstand harsh operational conditions.

Among the most advanced strategies, host–guest recognition stands out. PAMs modified with cyclodextrins (CDs) show significant thermal stability, exhibiting less than 20 % weight loss at 300 °C in an oxygen-free environment [2]. The interlocking effect of CDs with hydrophobic guest monomers produces a defined, sterically protected physical crosslink, forming a more compact and resilient three-dimensional network than simple hydrophobic associations. A striking case study by Strandman and Zhu (2016) [85] demonstrated a β-CD/cholic-acid system in which β-CD was grafted onto a poly(N,N-dimethylacrylamide) backbone (8 mol %) complexes with 2 mol % cholic-acid pendants to create fully reversible inclusion crosslinks. With a 12.5 wt% polymer and a 1:1 host–guest ratio, the hydrogel achieved its peak storage modulus (G′), and following shear-induced rupture, recovered its full G′ within 30 s, confirming rapid self-healing; competitive displacement by adamantane carboxylate validated the dynamic mechanism. A subsequent approach that pre-organized vinyl–functional β-CD and adamantane monomers prior to polymerization produced gels with G′ ≈ 680 kPa and ≈99% recovery of mechanical strength after 24 h. In stark contrast, conventional HPAM hydrogels typically exhibit G′ at the order of 1–10 kPa, recover less than 50 % of their modulus over hours, and suffer irreversible degradation under shear and temperatures above 90–100 °C [1,17], underscoring the superior mechanical robustness and rapid reconfiguration afforded by host–guest recognition.

### 6.2. Dynamic Covalent PAMs Based on Reversible Chemical Bonds

Dynamic covalent chemistry represents a distinct class of adaptive polymers that combines the strength of covalent bonds with the reversibility of supramolecular systems. These are defined by their ability to reversibly break and re-form under certain stimuli [2,86,87], allowing PAM-based systems to demonstrate self-healing capabilities and adapt to changing reservoir conditions without irreversible harm. A foundational example is the system described by Otsuka (2013) [81], where polymers containing alkoxyamine units undergo a thermally induced radical crossover reaction. At temperatures above 60–100 °C, the C-ON bonds reversibly cleave and reform, allowing initially separate polymer chains to become covalently crosslinked into a macroscopic gel. Crucially, this process is governed by chemical equilibrium and can be reversed by adding a small-molecule competitor, enabling the gel to be controllably dissolved back into a solution. This principle has been leveraged to create materials with extreme stability. A gel inspired by mussel adhesion proteins, which utilizes dynamic catechol-based chemistry, has been shown to maintain its mechanical integrity and self-healing properties at temperatures up to 200 °C [88]. This performance dramatically surpasses the operational limits of conventional HPAM (typically <100 °C) and most physically crosslinked supramolecular systems, establishing dynamic covalent chemistry as an essential strategy for the most demanding ultra-deep reservoir applications.

The collective integration of these supramolecular interactions confers significant and measurable advantages to PAM-based systems in extreme reservoir conditions, fundamentally differentiating them from, and substantially outperforming, conventional PAMs (summarized in Table 3). Supramolecular PAMs exhibit superior thermal and shear stability. Hydrogen bonding, hydrophobic associations, and dynamic covalent bonds facilitate molecular rearrangement and energy dissipation, preserving performance even at temperatures exceeding 180 °C and under high shear, a resilience that contrasts sharply with the rapid viscosity decline in conventional PAMs. Furthermore, these advanced systems demonstrate enhanced salt tolerance. Supramolecular interactions, particularly hydrophobic association and electrostatic forces, stabilize polymer conformation against the ‘salting-out’ effect, maintaining viscosifying power and stability in high-concentration brines. Also, the dynamic nature of supramolecular bonds enables adaptive and multifunctional behavior. These PAM systems respond precisely to reservoir stimuli (e.g., pH, temperature, magnetic fields), allowing for controllable gelation, degradation, or rheological changes. This multifunctionality extends to targeted delivery, self-healing, and synergistic contributions to IFT reduction and wettability alteration, capabilities beyond the scope of traditional PAMs. These attributes position supramolecular PAMs as a critical frontier for efficient, cost-effective, and environmentally responsible EOR in demanding subsurface environments.

## 7. Sustainable and Bio-Inspired Polyacrylamide-Based Systems

The escalating environmental concerns associated with conventional chemical EOR processes, primarily stemming from the non-degradability, potential toxicity, and high production costs of synthetic polymers, have spurred significant research into sustainable and bio-inspired alternatives. PAM, despite its broad utility, shares some of these environmental drawbacks, particularly its resistance to natural degradation. Addressing these limitations is paramount for ensuring the long-term viability and ecological compatibility of PAM-based materials in reservoir management. This section explores recent innovations in designing PAM systems that incorporate bio-derived components, promote biodegradability, or contribute to sustainable energy solutions, thereby aligning EOR practices with broader environmental goals.

### 7.1. Bio-Inspired Materials and Composites for Enhanced Performance

The integration of natural, renewable materials into PAM-based formulations is a crucial step towards sustainable EOR, enhancing performance while mitigating environmental concerns. These bio-inspired composites leverage biomass’s inherent structural and chemical diversity to develop multifunctional materials.

A notable advancement is lignin–polyacrylamide hydrogels, which utilize lignin, an abundant and cost-effective biopolymer from plant waste, to impart enhanced mechanical, thermal, and salinity resistance. Wang et al. (2025) [92] demonstrated that lignin-crosslinked PAM (L-cPAM) hydrogels achieved a 53.6% increase in single compressive strength (up to 180.71 kPa at 15% lignin dosage). This can be compared to the toughness of advanced double network (DN) hydrogels, which exhibit fracture energies (~1000 J/m^2^) nearly 100 times greater than brittle, conventional single-network gels (~10 J/m^2^) [22]. These hydrogels exhibited significantly improved thermal stability, with the initial thermal decomposition temperature rising from 150 °C to 181 °C, and maintained robust salinity resistance, showing minimal weight change (0.65% after 7 days) in highly saline brines (21 × 10^4^ mg/L TDS) at 150 °C. Beyond EOR, lignin–PAM hydrogels offer a sustainable solution for sand fixation, with Xu et al. [19] reporting a 90% reduction in sand production and up to 66.5% increased water retention in coreflooding tests. Their inherent slow degradation (mass loss between 16.4% to 24.9% over 90 days) ensures prolonged environmental efficacy.

Similarly, glycerol-reinforced PAM-PEI gels utilize glycerol, a sustainable byproduct, to enhance gel properties via extensive hydrogen bonding. Morais et al. (2023) [10] demonstrated that glycerol incorporation accelerated gelation by up to 30% and significantly delayed syneresis by 110%, contributing to superior gel stability. These gels showed considerably improved thermal stability, retaining approximately 50% of initial weight at 176 °C, a marked improvement over basic HPAM-PEI gels, which lost ~95% weight. Rheological analysis confirmed enhanced elastic properties with tan delta values below 0.1, signifying a robust elastic material. This approach offers precise density control, reducing the need for solid fillers in targeted plugging and abandonment applications.

### 7.2. Biodegradation of Polyacrylamide for Environmental Mitigation

Evaluating the enduring environmental consequences of synthetic PAM is essential for its responsible application. PAM, as a synthetic polymer, is typically viewed as resistant to quick biodegradation, resulting in its accumulation in the environment. However, recent studies investigate its potential for anaerobic biodegradation pathways. Li et al. (2024) [5] performed an in-depth investigation into the anaerobic biodegradation of anionic polyacrylamide (A-PAM) within oil sands tailings, an environmental matrix of significant importance. The findings indicated that A-PAM experienced considerable degradation, especially at lower concentrations (50–100 mg/kg TS), leading to a notable reduction in A-PAM concentration and molecular weight (with a Mw reduction of up to 76.2% and a Mn reduction of up to 80.2% at a 50 mg/kg TS A-PAM concentration). This process resulted in intermediate products like low-molecular-weight polyacrylic acids instead of the direct release of acrylamide monomer, and it also facilitated methane production, achieving a yield of up to 8.9 mL of methane. This suggests that particular microbial communities in anaerobic settings, such as Smithella, Candidatus_Cloacimonas, and DMER64, have the ability to decompose the A-PAM backbone, using it as a source of carbon and nitrogen [5]. Importantly, the degradation products showed low levels of acute toxicity and genotoxicity, addressing significant environmental issues related to PAM accumulation. The investigation revealed that elevated A-PAM concentrations may hinder biodegradation, underscoring the necessity of optimizing these concentrations for efficient degradation. This study provides a scientific foundation for reducing environmental risks and advancing the creation of more inherently biodegradable PAM derivatives for future enhanced oil recovery applications.

### 7.3. PAM Gels in Biogas Purification: A Green Energy Application

The use of PAM gels in biogas purification, while not a direct application in enhanced oil recovery, demonstrates their versatility and underscores their significance in wider sustainable energy efforts. This indirectly supports the sustainable properties of PAM materials in the energy sector. Biogas, a renewable fuel, contains impurities like CO_2_ and H_2_S that reduce its energy content and contribute to corrosion [93]. Sahoo et al. (2025) [16] investigated the application of PAM gels, created using chromium (III) acetate and piperazine, as a viable medium for the reduction of CO_2_ and H_2_S emissions. The findings indicated that optimized PAM gels achieved remarkable biogas purification efficiencies, reaching a maximum methane concentration of 90.1% and nearly complete H_2_S removal (down to 0–2 ppm). The gel displayed non-Newtonian, shear-thinning properties that improved gas absorption and management, while its robust structure allowed for regeneration and reusability. The optimal gel formulation exhibited remarkable equilibrium constants (K = 7.988 and 9.101) along with negative Gibbs free energy values (as low as −5526.68 J/mol), indicating a highly spontaneous and efficient CO_2_ absorption process. The capacity of PAM gels to specifically capture these impurities underscores their promise in delivering a cleaner and more sustainable fuel for diverse applications, aiding in the reduction in greenhouse gas emissions and environmental pollution. This application points out PAM’s role as an adjustable chemical platform, contributing not only to improved oil recovery, but also to several critical processes within the green energy sector. The progress in sustainable and bio-inspired PAM systems tackles the environmental impact and performance constraints of conventional synthetic materials. Through the integration of bio-derived components like lignin and glycerol, examination of PAM’s biodegradability, and novel applications in green energy, significant advancements are being made towards developing more sustainable and resilient solutions in reservoir management.

## 8. Integrated Reservoir Management Strategies and Practical Considerations

The complex nature of today’s petroleum reservoirs, marked by their heterogeneity, maturity, and difficult production environments, requires a move away from isolated interventions toward more integrated management approaches. Relying solely on either mechanical or chemical methods often falls short of achieving both optimal recovery and long-term wellbore stability. This section looks into comprehensive strategies that bring together different technologies, with a focus on hybrid sand control, preventing damage during conformance treatments, and the key role of advanced monitoring and prediction.

### 8.1. Chemical–Mechanical Hybrid Sand Control

Both chemical and mechanical sand control methods have their downsides. While chemical consolidation techniques are good at bonding sand grains and stopping particle movement, and mechanical tools like screens and gravel packs offer physical support, they are not without limitations [7]. Mechanical screens can easily become plugged or eroded. At the same time, in situ chemical treatments might not spread evenly, and they can harm permeability over time [94,95]. To avoid these problems, chemical-mechanical hybrid sand control methods have become popular because they combine the strengths of both approaches. A good example is pairing chemical sand stabilization with a squeeze gravel pack (SGP). An SGP involves injecting gravel or coarse sand into the area near the wellbore to provide mechanical support, which is particularly useful in zones where sand has already created voids [8]. This mechanical frame is then strengthened with chemicals by injecting stabilizers, typically resins or polymers, that bind the gravel together with the surrounding formation particles. This two-part strategy creates a strong, permeable zone that can handle the stress of fluid flow. Song et al. (2024) [8] investigated this approach, highlighting the necessity to optimize pumping parameters such as initial fluid loss (spurt loss) and injection rate. This ensures the chemical stabilizer penetrates thoroughly and distributes uniformly. Mörtl (2009) [6] presented the concept of controlled spurt loss, wherein a diverting agent, such as PAM gel particles or specific resins like FDP-S875, facilitates the initial entry of resin into the formation. It then forms a filter cake that pushes the rest of the resin to less permeable areas, ensuring a well-consolidated zone around the wellbore. Lab experiments by Song et al. (2024) [8] showed that chemical sand stabilization could change a “severe collapse” sanding pattern to a “wormhole sanding cavity” or “mild collapse,” which greatly reduced sand production (e.g., a 90% reduction using a PAM hydrogel from Marandi et al. [22]. Moreover, this combined approach resists erosion from production fluids better than mechanical support alone. For these hybrid methods to work well, it is important to choose chemical agents that are compatible with the carrier fluid, can handle reservoir conditions, and can be applied uniformly in complex, multilayered reservoirs.

### 8.2. Damage Prevention and Remediation in Conformance Control

A long-standing problem with using polymer gels for deep conformance control is the risk of unintentionally damaging desirable, low-permeability oil-bearing zones. When injected into diverse reservoirs, low-viscosity gelant solutions can seep into these unswept zones and gel up, causing unintended plugging and reducing oil mobility. This ultimately undermines the overall EOR effort. To deal with this critical issue, protective treatments that use gel-breaking agents have been created. These agents are made to break down the polymer gel network after it has performed its main job of diverting flow or plugging a zone, especially in areas where it might impede future oil production. Ref. [20] looked into using ammonium persulfate as a gel-breaker for in situ cross-linked PAM gels. They found that an optimal concentration of ammonium persulfate (2–5%) not only broke down the gel quickly with minimal residue, but also effectively reversed the damage. They used nuclear magnetic resonance (NMR) T2 spectrum analysis to track how fluids were distributed and how well the treatment worked at the pore scale. The results revealed that after injecting ammonium persulfate into diverse reservoir cores, the gel damage on the face of the low-permeability layers was relieved. This was seen in the NMR T2 signals shifting from low-permeability zones back toward medium–low-permeability areas. This shift pointed to the re-activation of previously unswept oil and a better sweep efficiency, which led to an incremental oil recovery of 18.75% OOIP in oil-saturated cores [20]. Such “damage-preventive methods” are vital to make sure that conformance treatments divert flow as intended without harming the productivity of bypassed oil zones, especially in mature oilfields.

### 8.3. Monitoring and Prediction Technologies for Optimized Reservoir Management

Managing reservoirs effectively, particularly for complex EOR and sand control operations, depends heavily on accurate, real-time monitoring and predictive models. These technologies help operators make smart decisions, fine-tune treatment designs, and respond to changing reservoir conditions.

Artificial intelligence (AI) and machine learning (ML) techniques are being used more and more to predict and manage sand production. By sifting through huge datasets from historical production, well logs, and field measurements, AI algorithms can spot subtle patterns that signal upcoming sand problems, predict sand production rates, and optimize control treatments [96]. These models can learn from past successes and failures to suggest the best strategies for specific well conditions. For example, Ref. [8] mentioned using their own software with integrated ML to simulate composite sand control designs and predict outcomes, which allows for precise SGP design optimization based on previous sanding behavior.

Beyond just predicting outcomes, advanced characterization techniques give us crucial information about how well treatments are working downhole. For example, nuclear magnetic resonance (NMR) T2 spectroscopy provides a non-destructive, pore-scale insight into how fluids are distributed and how much residual oil is left in reservoir cores [20]. It lets researchers see how polymer gels or nanoparticles affect fluid flow in different permeability layers, track how far a gel has propagated, and confirm if gel-breaking treatments have worked. Similarly, X-ray computed tomography (CT scan) gives a 3D picture of what is happening inside a core and how fluids are distributed. Ref. [61] used CT scans to directly see how Fe_3_O_4_ nanoparticles lined up into “columnar structures” when stimulated by a magnetic field, which confirmed their role as “microscopic pistons” for pushing out more oil. This kind of technique is invaluable for understanding how new hybrid materials, like magnetic gels, interact with the reservoir rock and affect fluid flow in real time [61]. Finally, molecular dynamics (MD) simulations, though they require a lot of computing power, provide atom-level insights into the basic interactions (like how polymers stick to calcite or the tensile strength of consolidated rock) that control what we see on a larger scale [97]. These simulations let us screen chemicals and understand mechanisms that are hard to observe in experiments, helping to inform the design of the next generation of materials. When all these monitoring and prediction technologies are brought together into a complete reservoir management plan, they provide the data and analytical power needed to customize treatments, prevent early failures, cut costs, and obtain the most oil possible from increasingly complex and difficult oilfield environments.

## 9. Key Properties, Characterization Techniques, and Mechanistic Insights

Understanding the performance of PAM-based systems in intricate reservoirs necessitates a multiscale characterization strategy, connecting molecular-level interactions to macroscopic, core-scale behavior. A comprehensive array of sophisticated methods is utilized to achieve an in-depth understanding, guiding the logical design and forecasting models of these materials.

At the molecular and chemical level, various spectroscopic and analytical methods are essential. X-ray diffraction (XRD) is used to identify crystalline phases and verify the successful integration of nanoparticles or crosslinkers into the polymer matrix [5,21,67]. Fourier-transform infrared (FTIR) spectroscopy provides crucial insights into chemical structure by analyzing functional groups, allowing researchers to track reactions such as cross-linking, the confirmation of polymer functionalization, and the quantification of hydrolysis by observing changes in amide absorbance [29,59]. The evaluation of surface properties of dispersed components is conducted through zeta potential measurements, which quantify surface charge and are essential for comprehending colloidal stability and adsorption behavior.

When examining the micro- and nano-scale structure, the importance of electron microscopy cannot be overstated. Scanning electron microscopy (SEM) provides intricate visualization of surface morphology, enabling the differentiation between porous and compact gel structures and offering insights into degradation processes [5,22]. To achieve higher resolution, transmission electron microscopy (TEM) is employed to observe internal nanostructures, including the core–shell architecture of engineered nanoparticles [59]. The measurement of nanoparticles’ size and stability in solution is conducted through dynamic light scattering (DLS), which assesses their hydrodynamic diameter and aggregation state.

The large-scale characteristics of these systems are measured using various methods. The rheological profile, encompassing viscosity and viscoelastic moduli (G′, G″), is assessed utilizing Rheometers via dynamic oscillatory and steady shear measurements [16,17]. Thermal stability is mainly evaluated using thermogravimetric analysis (TGA), which monitors mass loss in relation to temperature to identify the onset of degradation and analyze material composition [2,22]. Contact angle goniometry is employed to directly measure interfacial phenomena for wettability alteration, while interfacial tensiometry is utilized to quantify interfacial tension reduction [23,24].

Ultimately, the core-scale performance is depicted in a non-invasive manner. X-ray-computed tomography (CT scan) delivers three-dimensional images that illustrate the arrangement of materials and the distribution of fluids within reservoir cores, validating the depth of gel penetration and even allowing for visualization of the alignment of magnetic nanoparticles [61]. Nuclear magnetic resonance (NMR) T2 spectroscopy provides detailed insights at the pore scale regarding fluid distribution, the mobilization of residual oil, and the effectiveness of gel degradation treatments [20]. In addition to these experimental techniques, molecular dynamics (MD) simulations offer exceptional atomistic-scale insights into polymer–rock adsorption, intermolecular forces, and the essential mechanisms that dictate macroscopic performance [97].

The combined multidisciplinary characterization efforts, outlined in Table 4, facilitate a thorough understanding of PAM-based systems, encompassing their molecular design and field-scale potential.

## 10. Challenges and Future Outlook

Even with significant laboratory advancements, bringing next-generation PAM systems to widespread field application is fraught with persistent challenges. Moving these innovative materials from concept to the reservoir requires not only solving inherent material limitations, but also breaking through critical technical bottlenecks in modeling, monitoring, and sustainable engineering. A truly forward-looking review, however, must go beyond listing problems, and instead connect them to actionable research pathways, clarifying the specific hurdles that future research must prioritize [3,4,59].

A primary challenge for nanoparticle-enhanced systems remains colloidal stability. The tendency of nanoparticles to agglomerate in high-salinity brines can lead to a loss of function and potential formation damage. Promising solutions lie in advanced surface functionalization strategies, such as grafting steric stabilizers like polyethylene glycol brushes onto the nanoparticle surface or developing robust, salt-tolerant core–shell architectures. This issue of predictability extends to multicomponent systems, where complex interactions like chromatographic separation can impede reliable performance [21,23,24,78]. A key path forward is the development of “unitized” multifunctional polymers, where different functionalities are covalently linked onto a single terpolymer backbone, preventing separation during transport. Finally, the balance between performance and cost must be addressed. A critical solution pathway lies in utilizing low-cost, sustainable feedstocks. The successful application of lignin and glycerol in high-performance gels provides a clear precedent, suggesting that future work should focus on efficient catalytic methods to convert biomass into the high-value functional monomers needed for these advanced polymers.

Beyond designing the materials themselves, several overarching technical bottlenecks must be solved to unlock their full potential. The first, and arguably most significant, is the multiscale modeling gap. The field currently uses siloed modeling approaches: molecular dynamics (MD), pore-scale/CFD models, and field-scale reservoir simulators. The challenge is not a lack of models at each scale, but rather the absence of a robust methodology to pass information seamlessly between them. A major breakthrough will require developing AI-driven, multiscale digital twins to enable highly accurate simulation and autonomous optimization of complex operations and material design [20,97]. Thinking along these lines, the future design of these complex systems can also draw inspiration from synergistic composite materials in adjacent fields. A compelling case study by Zheng et al. [98], for example, demonstrated that a biochar support in a MOF composite acted as an electron carrier and that engineered lattice defects created new active sites, a design philosophy directly transferable to creating next-generation PAM–nanoparticle hybrids with engineered synergistic interfaces.

A second major bottleneck is the gap between laboratory certainty and reservoir uncertainty: how can we verify what a polymer is actually doing thousands of feet underground? This lack of in situ monitoring is a critical hurdle, particularly for precisely placing treatments in heterogeneous reservoirs without inadvertently damaging low-permeability zones [1,20]. A key area for future research is, therefore, the development of advanced downhole sensing and tracer technologies. This could involve incorporating environmentally benign fluorescent tags, like quantum dots, into the polymer backbone to monitor degradation via produced fluids. Another frontier is leveraging distributed fiber-optic sensing (DAS/DTS) to infer changes in fluid rheology in situ, providing real-time feedback that enables adaptive control for dynamic field optimization [3,8].

**Figure 10:**
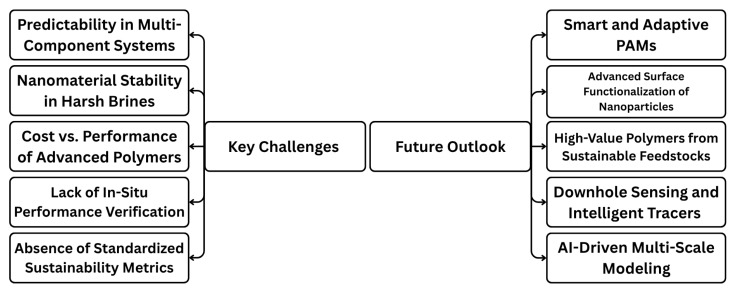
Challenges and future outlook of next-generation PAM-based systems in reservoir management.

Finally, as the field rightly moves towards ‘sustainable’ and ‘bio-inspired’ solutions, a third bottleneck emerges: the lack of standardized sustainability metrics to define what ‘sustainable’ truly means in practice. To address this, the academic and industrial communities must collaborate to establish a framework for life-cycle assessment (LCA) specifically for EOR chemicals. This would require standardized protocols to evaluate everything from the carbon footprint of monomer synthesis, especially from biomass, to the ecotoxicity of the polymer and its degradation products, addressing the environmental and regulatory transparency concerns [5,19]. Adopting Green Chemistry metrics, such as process mass intensity (PMI), for polymer manufacturing will be crucial for transparently comparing the sustainability of new materials against conventional ones. By focusing research efforts on these specific solutions and technical bottlenecks including the development of smart, multistimuli responsive PAMs [2,59,88], the field can accelerate the transition of these promising laboratory innovations into reliable, cost-effective, and sustainable field-scale technologies. Figure 10 provides a visual summary of these challenges and potential research direction.

## 11. Conclusions

This review has comprehensively synthesized the remarkable evolution of polyacrylamide (PAM)-based systems, transforming them from conventional EOR and sand control agents into multifunctional materials for challenging reservoir environments. We have elucidated how the strategic integration of nanomaterials significantly enhances rheological properties, interfacial phenomena, and stability [23,24,59,61]. Concurrently, the advent of supramolecular chemistry, leveraging dynamic non-covalent interactions, has imbued PAM-based systems with unprecedented resilience, self-healing capabilities, and adaptability to extreme temperatures and salinities [2,10,19,88].

This synthesis underscores a critical shift towards integrated reservoir management, combining advanced chemical designs with mechanical strategies for precise sand control and conformance [8,9]. Crucially, the increasing focus on sustainability, exemplified by bio-inspired materials and enhanced biodegradation pathways, aligns PAM technology with pressing environmental demands. While challenges in scalability and full predictability persist, PAM, continually reinvented through cutting-edge chemistry and holistic strategies, is poised to remain an indispensable and increasingly sustainable cornerstone for maximizing hydrocarbon recovery in the complex energy landscape of the future.

## Figures and Tables

**Figure 6:**
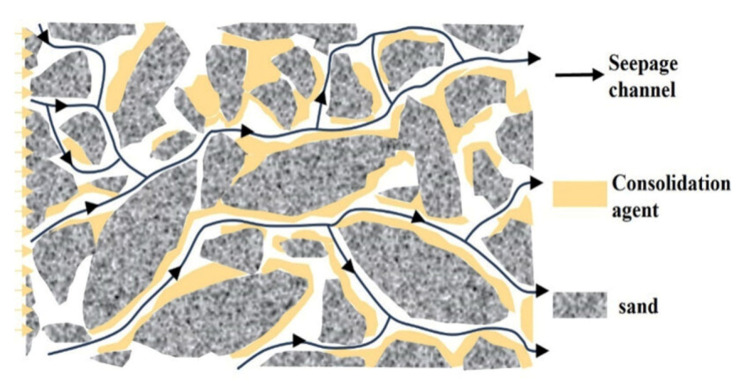
Sand consolidation agent’s effect in unconsolidated sand [58].

**Table 1:** Classification, properties, and applications of major polyacrylamide derivatives.

Derivative	Key Functional Group(s)	Characteristic Properties	Applications	References
Non-Ionic Polyacrylamide (Parent Polymer) NPAM	Amide (-CONH_2_)	Neutral charge; minimal interaction with salts Base for further chemical modification Effective flocculant in low-ionic media	Friction reduction in freshwater fracturing Mineral processing	[35,36]
Anionic/Partially Hydrolyzed Polyacrylamide APAM/HPAM	Carboxylate (-COO^−^Na^+^) Amide (-CONH_2_)	Anionic charge provides chain extension and high viscosity in fresh water High sensitivity to salinity and divalent ions	Polymer flooding (EOR) Friction reduction Drilling fluid additive	[1,26,28]
Cationic Polyacrylamide CPAM	Quaternary ammonium (-N^+^R_3_Cl^−^) Amide (-CONH_2_)	Cationic charge Strong affinity for negatively charged clays and silica Powerful flocculant and charge neutralizer	Clay stabilization in sandstone formations Oily wastewater treatment Sludge dewatering	[37]
Hydrophobically Modified Polyacrylamide HMPAM/HAPAM	Hydrophobic groups (e.g., CₙH_2_ₙ₊_1_) Anionic/non-ionic backbone	Forms intermolecular associations in water Improved viscosity and stability in high-salinity brines Enhanced shear resistance	High-salinity EOR Chemically tuned fracturing fluids Conformance control gels	[32,34,38]
Sulfonated Polyacrylamide (AM/AMPS Copolymer)	Sulfonate (-SO_3_^−^) Amide (-CONH_2_)	Strong anionic charge (sulfonate) Excellent thermal stability (>120 °C) Superior tolerance to divalent ions (Ca^2+^, Mg^2+^) due to steric hindrance	High-temperature/high-salinity (HTHS) EOR HTHS fracturing fluids HTHS drilling fluid additives116921	[17,30,31]

**Table 2:** Summarization of the comprehensive case study analysis of nanoparticle-enhanced PAM systems.

Author and Year	Material	Case Study/Innovation	Technical Details and Key Findings	Application
[59]	Fe_3_O_4_@PEI/PAM Smart Polymer Gel	Magnetic smart polymer gel: core–shell architecture enabling magnetic controllability and robust crosslinking.	The system, with NP size of 3.83 nm and density of 2.98 g/cm^3^, remained solid at 120 °C. Its mechanical qualities included 25,490 Pa storage and 7610 Pa loss modulus. After 1 month at 120 °C, it obtained 126,780 mPa·s gelation strength, 8.56 MPa bearing capacity, and 97.83% plugging efficiency in core displacement trials, increasing water flow fractional enhancement from 6.3% to 97.2%.	Magnetically controllable directional plugging for conformance control in high-temperature oil reservoirs; breakthrough in intelligent reservoir management.
[63]	PAM + Functionalized Magnetite Nanoparticles (Fe_3_O_4_)	Polyacrylamide–magnetite nanocomposite hydrogels: covalent integration of functionalized NPs as inorganic crosslinkers for enhanced mechanical properties.	Microstructural examination (SEM, HR-TEM) revealed the homogeneous distribution of 5–14 nm spherical Fe_3_O_4_ NPs, producing a hybrid organic–inorganic network. This improved mechanical qualities over traditional polymer gels.	Soft tissue engineering and biomedical scaffolds (potential for EOR application via similar strengthening principles).
[67]	AM/AMPS Co-Polymer Gels Crosslinked with PEI + Nylon Fiber	Temperature-resistant gel systems: development of robust gels for extreme high-temperature and high-salinity conditions.	Optimized with 1.0% AM/AMPS polymer, 0.1% PEI crosslinker, and 0.5% nylon fiber, these gels achieved H-level strength and excellent thermal stability. Long-term tests showed a syneresis rate < 2.5% (after 120 days at 240,720 mg/L salinity) and stability up to 130 °C. Demonstrated 94% plugging efficiency in sand-filled pipe experiments.	Enhanced oil recovery in harsh (high-temperature/high-salinity) environments.
[59]	PEI@Fe_3_O_4_@PEI Nanosheets in PAM-PEI Gel Networks	Magnetic nanoparticle-enhanced gel stability: modulating syneresis rate and gel strength through magnetic nanoparticle chelation.	Incorporating PEI@Fe_3_O_4_@PEI nanosheets, the gels showed enhanced thermal stability, with decomposition temperature increased to 198.45 °C, and improved weight retention by 25.85% compared to conventional systems. Magnetic responsiveness enabled directional control.	High-performance polymer gels with improved stability and responsiveness.

**Table 3:** Comparative performance of adaptive PAM network systems in harsh environments.

Class	Interaction Type	Mechanism of Enhancement	Comparative Performance vs. Conventional PAM	Experimental Validation
Supramolecular (Physical Cross-Links)	Hydrophobic Association and Hydrogen Bonding	Aggregation of nonpolar segments and H-bonds form a robust, energy-dissipating physical network.	Provides high mechanical toughness and thermal stability, whereas conventional gels are brittle and thermally unstable.	- Withstands compressive stress of 3.82 MPa at 88.3% compression; stable at 135 °C; plugging rate > 95% [82,89]. - Double-tail hydrophobic PAM maintained > 50 mPa·s at 150 °C under 170 s^−1^ shear [90], significantly outperforming conventional PAMs that undergo rapid degradation.
	Electrostatic Interactions	Coulombic forces between oppositely charged polymer chains form a dynamic, physically cross-linked network.	Achieves higher viscosity at lower concentrations and exhibits superior shear resilience compared to conventional PAMs, which require higher concentrations and show poorer recovery after shear.	0.4 wt% supramolecular system achieves viscosity of a 0.6 wt% conventional polymer; retains 73.3% viscosity after shear vs. 53.5% for conventional [83].
	Host–Guest Recognition	Cyclodextrins (hosts) encapsulate hydrophobic guests, forming highly stable, rapidly reversible inclusion complexes.	Imparts exceptional thermal stability and rapid, autonomous self-healing, in contrast to conventional hydrogels which are not self-healing, mechanically weak (G′ ≈ 1–10 kPa), and degrade >90 °C.	- G′ ≈ 680 kPa (vs. <10 kPa for HPAM); recovers full strength in 30 s after shear rupture; stable at 300 °C (<20% weight loss) [85]. - CD-modified PAM maintained high viscosity after shearing at 120 °C (Poly-A at 188.4 mPa·s, Poly-B at 232.2 mPa·s), far outperforming conventional HPAM (~50 mPa·s) [37].
Dynamic Covalent (Reversible Chemical Bonds)	Dynamic Covalent Bonds	Reversible cleavage and reformation of covalent bonds (e.g., catechol chemistry) in response to a specific stimulus.	Achieves extreme thermal stability and stimulus-gated self-healing, overcoming the irreversible molecular scission and complete loss of function seen in conventional PAMs under high stress.	Mussel-inspired gels maintain robust mechanical performance and self-healing capabilities at temperatures up to 200 °C [88]. Self-healing efficiency of transesterification vitrimers: >80% [91].
Supramolecular Reinforced Chemical Gel	Supramolecular + Covalent Cross-Links	Supramolecular forces provide initial viscosity and shear resistance, followed by chemical crosslinking.	Enables ultra-high temperature resistance at significantly lower polymer and cross-linker concentrations than conventional gels, which require much higher loadings for similar performance.	Final viscosity of 72.35 mPa·s after 2 h at 200 °C using only 0.4 wt% polymer [83].

**Table 4:** Summary of key characterization techniques and insights gained.

Characterization Technique	Principle/Measured Property	Key Insight Gained for PAM Systems
Rheometry	Viscosity, storage/loss moduli (G′/G″), Tan Delta	Fluid injectivity and mobility control; viscoelasticity (elasticity, syneresis prevention).
Thermogravimetric Analysis (TGA)	Mass change with temperature	Polymer thermal stability; degradation onset/profiles; compositional analysis.
Nuclear Magnetic Resonance (NMR) T2 Spectroscopy	Relaxation time of fluids	Fluid distribution and flow paths; gel/polymer degradation; residual oil mobilization.
X-Ray-Computed Tomography (CT Scan)	3D internal structure, X-ray absorption differences	Gel/NP placement and plugging; 3D fluid/material distribution; magnetic responsiveness.
Scanning Electron Microscopy (SEM)	Surface morphology, microstructure	Micro-scale material structure; inter-particle interactions.
Transmission Electron Microscopy (TEM)	Nanostructure/morphology (internal)	Nanoparticle morphology; core–shell structures.
Dynamic Light Scattering (DLS)	Particle size, hydrodynamic diameter	Dispersion stability; aggregation behavior.
X-Ray Diffraction (XRD)	Crystallinity, chemical phases	Material composition; network formation confirmation.
Fourier-Transform Infrared (FTIR) Spectroscopy	Functional groups, molecular interactions	Molecular bonds; functional groups; reaction confirmation.
Zeta Potential	Surface charge, colloidal stability	Surface charge; colloidal stability; adsorption behavior.
Contact Angle Goniometry	Wetting angle	Rock wettability alteration; fluid–surface affinity.
Interfacial Tensiometry (IFT Tensiometry)	Interfacial tension (IFT)	IFT reduction efficacy; oil mobilization.
UV-Vis Spectrophotometry	Light absorption by chemical species	Chemical concentration quantification.
Molecular Dynamics (MD) Simulations	Atomistic-level modeling of interactions	Atomistic-level material properties; interfacial mechanisms.

## Data Availability

Data sharing is not applicable. No new data were created or analyzed in this study.

## Nomenclature

cp	Centipoise (unit of dynamic viscosity)
mL	Milliliter (unit of volume)
g	Gram (unit of mass)
mPa·s	Millipascal-second (unit of dynamic viscosity)
G′	Storage modulus (unit: Pascal, Pa)
mN/m	Millinewtons per meter (unit of surface tension)
G″	Loss modulus (unit: Pascal, Pa)
nm	Nanometer (unit of length)
J/mol	Joules per mole (unit of energy)
Pa	Pascal (unit of pressure/stress)
K	Equilibrium constant (dimensionless)
ppm	Parts per million (unit of concentration)
kPa	Kilopascal (unit of pressure)
psi	Pounds per square inch (unit of pressure)
Mw	Weight-average molecular weight
PV	Pore volume (dimensionless)
Mn	Number-average molecular weight
s^−1^	Reciprocal seconds (unit of shear rate/frequency)
mD	Millidarcy (unit of permeability)
wt%	Weight percent (unit of concentration)
mg/kg	Milligrams per kilogram (unit of concentration)
%	Percent (dimensionless)
mg/L	Milligrams per liter (unit of concentration)
µm	Micrometer (unit of length)
Acronyms	
AI	Artificial intelligence
ML	Machine learning
A-PAM	Anionic polyacrylamide
mMWCNT	Modified multiwalled carbon nanotubes
ASL-PVA	Amphoteric lignin-based poly(vinyl alcohol)
NMR	Nuclear magnetic resonance
ATBS	Acrylamido-tertiary-butyl sulfonic acid
NPs	Nanoparticles
CDG	Colloidal dispersion gels
OOIP	Original oil in place
CDs	Cyclodextrins
PAM	Polyacrylamide
CFD	Computational fluid dynamics
PEI	Polyethyleneimine
CT	Computed tomography
PDI	Polydispersity index
DEM	Discrete element method
PPG	Preformed particle gels
DPR	Disproportionate permeability reduction
S-S	Disulfide bonds
EOR	Enhanced oil recovery
SGP	Squeeze gravel pack
FTIR	Fourier-transform infrared spectroscopy
SEM	Scanning electron microscopy
HPAM	Partially hydrolyzed polyacrylamide
SP	Surfactant–polymer
HQ	Hydroquinone
SMRF	Sulfomethylated resorcinol–formaldehyde
HMTA	Hexamethylenetetramine
TGA	Thermogravimetric analysis
IFT	Interfacial tension
TDS	Total dissolved solids
L-cPAM	Lignin-crosslinked polyacrylamide
TEM	Transmission electron microscopy
LSW	Low-salinity water
XRD	X-ray diffraction
